# Identification of phenocopies improves prediction of targeted therapy response over DNA mutations alone

**DOI:** 10.1038/s41525-022-00328-7

**Published:** 2022-10-17

**Authors:** Hamza Bakhtiar, Kyle T. Helzer, Yeonhee Park, Yi Chen, Nicholas R. Rydzewski, Matthew L. Bootsma, Yue Shi, Paul M. Harari, Marina Sharifi, Martin Sjöström, Joshua M. Lang, Menggang Yu, Shuang G. Zhao

**Affiliations:** 1 Department of Human Oncology, Madison, WI 53792 USA; 2 Department of Biostatistics and Medical Informatics, Madison, WI 53792 USA; 3 Department of Medicine, University of Wisconsin-Madison, Madison, WI 53792 USA; 4 Department of Radiation Oncology, UCSF, San Francisco, CA USA; 5 William S. Middleton Memorial Veterans Hospital, Madison, WI 53792 USA

**Keywords:** Molecular medicine, Predictive markers

## Abstract

DNA mutations in specific genes can confer preferential benefit from drugs targeting those genes. However, other molecular perturbations can “phenocopy” pathogenic mutations, but would not be identified using standard clinical sequencing, leading to missed opportunities for other patients to benefit from targeted treatments. We hypothesized that RNA phenocopy signatures of key cancer driver gene mutations could improve our ability to predict response to targeted therapies, despite not being directly trained on drug response. To test this, we built gene expression signatures in tissue samples for specific mutations and found that phenocopy signatures broadly increased accuracy of drug response predictions in-vitro compared to DNA mutation alone, and identified additional cancer cell lines that respond well with a positive/negative predictive value on par or better than DNA mutations. We further validated our results across four clinical cohorts. Our results suggest that routine RNA sequencing of tumors to identify phenocopies in addition to standard targeted DNA sequencing would improve our ability to accurately select patients for targeted therapies in the clinic.

## Introduction

Over the last decade, targeted therapies against a large range of oncogenic pathways have emerged as valuable additions to our anti-cancer armamentarium^1,2^. Targeted therapies have demonstrated particular success in patients harboring specific driver mutations, usually in their respective targets^3,4^. The FDA has approved EGFR inhibitors in *EGFR*-mutant NSCLC^5–7^, BRAF inhibitors in both *BRAF*-mutant melanoma^8,9^ and NSCLC^10^ PI3K inhibitors in *PIK3CA*-mutant breast cancer^11^ and PARP inhibitors in Homologous Recombination Deficient (HRD) ovarian^12^ and prostate^13^ cancer.

For many of the genes with FDA approved biomarker indications, there are frequently known hotspot mutations, such as the V600 mutations in *BRAF*^14^. Although the presence of these driver mutations tend to be informative for identifying patients for targeted therapies, there are often mutations of unknown significance which fall elsewhere in the gene that may or may not convey sensitivity^15^. Thus, the response in patients who harbor these mutations is not uniform, and many patients fail to respond even though they carry the driver mutation of interest^16–20^. Additionally, others lacking a mutation may still show benefit from treatment. The reasons for the variability in response are multi-factorial. First, not all mutations alter the function of the protein and different mutations can have wildly different phenotypic impacts depending on the location and amino acid change. Second, regulation via epigenetic, post-transcriptional, and post-translational changes can modulate the impact of mutations and lead to incomplete penetrance of the expected phenotype. Finally, there may be other modes of activation for a particular oncogenic pathway upstream, downstream, or even in a different pathway independent of mutations in the target itself.

The activation of many oncogenic pathways leads to distinct transcriptomic changes. However, to date, work assessing gene expression patterns mimicking DNA alterations has been limited in scope to specific targets or cancer types. We hypothesized that gene expression signatures that identify phenocopies of alterations in key DNA alterations would improve predictions of response and resistance to targeted therapies. For example, these signatures could identify additional tumors which phenocopy *EGFR*-mutant tumors that would respond to anti-EGFR therapy, without necessarily carrying an *EGFR* mutation. Likewise, these phenocopy signatures could also identify tumors with an *EGFR* mutation of unknown significance that do not display the *EGFR*-mutant phenocopy, and do not respond to anti-EGFR therapy. Herein, we developed phenocopy signatures of mutations in key cancer genes on 9248 patient samples across cancer types and validate in 1982 cell line experiments across three datasets. We found that these signatures improved our ability to predict response to targeted therapies compared to DNA mutations alone. We also demonstrated that these phenocopy signatures predict response in clinical cohorts and shift under the selective pressure of treatment. Unlike most of the previous literature in this area^21–33^, we do not directly train models to predict drug response. Instead, the association of drug response to our phenocopy signatures arise as an indirect but intended side effect.

## Results

### Model design

**Fig. 1:**
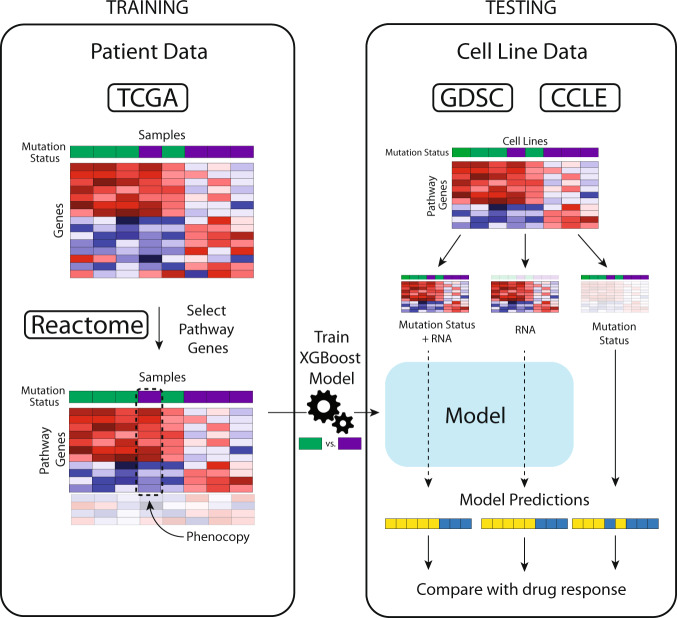
Schematic of phenocopy signatures model. For each gene of interest, known mutation status is determined (green – mutation; purple – no mutation) and an XGBoost model is trained on mutation status based off gene expression of the pathway of interest and the phenocopy signatures are locked (Training, left). Next, gene expression data from cell line databases or clinical studies are input into the phenocopy signatures which outputs a predicted phenocopy status. This phenocopy status is then compared to known DNA mutations for predicting drug response.

We first sought to define expression-based “phenocopy” signatures for various DNA mutations in therapeutically actionable pathways in cancer (Fig. 1). We designed the phenocopy signatures to identify RNA expression patterns of mutated tumors. The underlying assumption is that mutations in key oncogenic driver genes will be pathogenic more often than not, and that these pathogenic mutations will result in a somewhat uniform set of downstream transcriptional changes. Machine learning models such as XGboost do not require perfect information, and can learn these patterns even in the face of noise in the data (i.e. even with some non-pathogenic passenger mutations). To build these phenocopy signatures, we utilized publicly available data from TCGA, which contains mutation status and RNA expression data for over 11000 tumor samples across 33 different tumor types. For each actionable gene, an XGBoost model was trained using gene expression profiles of pan-cancer tumor samples paired with the known DNA alteration status. Each model was then trained to define a gene expression signature for eight different targetable pathways (EGFR, BRAF, PI3K-AKT, PARP/HRD, ERBB2, mTOR, JAK, and MAPK). To assess if the phenocopy signatures could predict drug response, independent data from the GDSC, CCLE and DepMap datasets were used which contain gene expression, DNA mutations, and drug responses across 969, 917, and 578 cancer cell lines, respectively. Additionally, we analyzed four clinical studies which have gene expression and treatment data for patients treated with a drug targeting one of the pathways listed above.

### Phenocopy signature predictions

After assigning a predicted alteration status to each cell line in the testing set with the XGBoost-driven model as described above, we investigated how many cell lines in our validation cohorts were marked as altered by the phenocopy signature alone, the DNA mutation status alone, or by both. DNA alterations were additionally split into mutations which have a known or predicted deleterious or pathogenic effect and those with unknown significance (Supplementary Fig. 1). Our goal was not to create signatures that would perfectly predict cell lines’ alteration statuses, as this would not offer additional insights. Instead, we created our phenocopy signatures so they would identify cell lines that phenotypically mimicked gene expression patterns of altered cell lines, whether or not they carried a canonical driver mutation. For all pathways, we found discordance between actual DNA mutation status and phenocopy predictions which suggests that there is additional information from the phenocopy signatures that may help inform drug response predictions.

Diverse tumor types are well represented across the cell line validation cohorts (Supplementary Figure 2). We next sought to understand the impact of CNV in the genes in each pathway. We clearly observed that CNV changes in the pathway are more frequent in the samples that have a phenocopy expression profile without a mutation (Supplementary Fig. 3). Since CNV influences gene expression, this suggests a potential mechanistic explanation for why these samples are demonstrating a gene expression phenocopy in the absence of a mutation in a driver gene. When we performed a similar analysis examining mutations instead of CNV, the differences were much less pronounced, again supporting the role of CNV in influencing the phenocopy gene expression patterns (Supplementary Fig. 4).

### Phenocopy signatures improve pan-cancer drug response predictions across multiple pathways

**Fig. 2:**
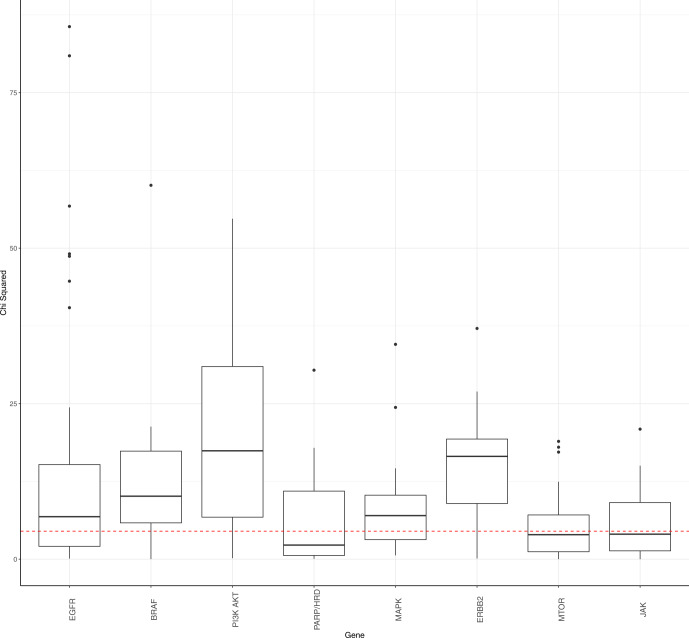
Phenocopy signatures significantly add to DNA mutations in predicting drug response across oncogenic pathways. Linear models for drug response were used to assess how much the phenocopy signatures added to DNA mutations across pathways. Each data point used in the boxplot represents a model for a single drug in a dataset. A larger *χ*^2^ value represents a more significant contribution of the phenocopy signature to DNA mutations. Red dotted line represents a significant FDR threshold of 0.05. Boxplots show median values along with the interquartile range.

Next, we assessed how our gene-expression based phenocopy signatures performed in adding predictive information on targeted therapy drug response compared to DNA alterations alone. To assess if the discordance between actual DNA mutation status and the phenocopy signature predictions improves predictions of drug response, we chose to assess eight different pathways: four of which have clinically actionable mutations in various cancer types (BRAF^8–10^, BRCA^13,34,35^, EGFR^5–7^, and PIK3CA^11^) and four of which are targets of ongoing research, but do not yet have FDA-approved indications (MAPK^36,37^, ERBB2^38,39^, mTOR^40^, and JAK^41^). We next tested if the phenocopy signatures improved the ability to predict drug response for drugs targeting these pathways. To accomplish this, we examined linear models of drug response to treatment targeting each pathway in the independent GDSC, CCLE, and DepMap cohorts, with both the true DNA alteration status and the phenocopy signatures as independent variables. To assess significance, a multiple-testing FDR-corrected chi-squared statistic was calculated for each drug/gene combination to determine if the addition of the phenocopy signature to DNA alterations alone improved the ability to predict drug response. Overall, model performance was significantly improved in 68% of cases across 165 different therapies targeting these eight pathways (Fig. 2). For 61% of drugs targeting EGFR, 75% of drugs targeting BRAF, 80% of drugs targeting PI3K-AKT, 50% of drugs targeting PARP/HRD, 64% of drugs targeting MAPK, 90% of drugs targeting ERBB2, 53% of drugs targeting mTOR, and 50% of drugs targeting JAK, the phenocopy signatures significantly added to DNA mutations alone.

**Fig. 3:**
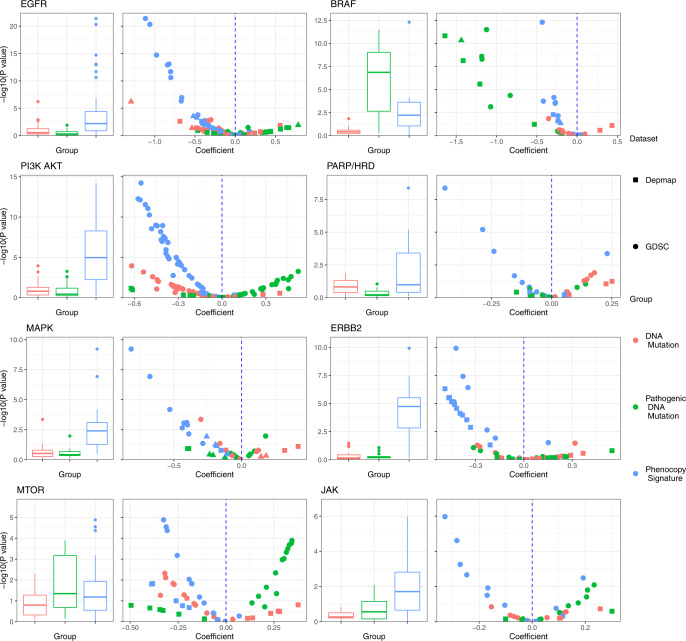
Detailed comparison of phenocopy signatures to DNA mutations. Linear models for drug response were used to assess how much the phenocopy signatures added to DNA mutations across pathways. Each model of a single drug is represented by three points, one for each independent variable (DNA mutation, pathogenic mutation, and phenocopy signature). The *x*-axis represents the linear coefficient, and the *y*-axis is the associated -Log_10_(*p*-value) of each independent variable in each linear model. Pathogenic mutations are mutations which are annotated as pathogenic by ClinVar or computational tools. Negative coefficients represent expected estimates, where the actual mutation status or predicted mutation status from the phenocopy signature is associated with increased sensitivity to the drug. Data points in the upper-left quadrant therefore represent drugs for which the phenocopy signature most significantly contributed to predicting drug sensitivity. Boxplot center line = median value, bounds of box (hinges) = interquartile range (IQR), bounds of whiskers = maximum of 1.5X IQR from the hinges.

We next sought to further examine the individual pathways and drugs in more detail. Volcano plots of the contributions of the phenocopy signatures, DNA mutations, and pathogenic mutations in the linear models redemonstrated how the phenocopy signatures added to DNA mutations for drugs targeting pathways with and without mutations as FDA indications (Fig. 3, top four panels and bottom four panels, respectively). Of note, negative coefficients represent expected estimates, where the actual mutation status or predicted mutation status from the phenocopy signature is associated with increased sensitivity to the drug. *BRAF* pathogenic mutations in particular successfully predicted response to BRAF inhibitors even after taking into account the phenocopy signatures, though the phenocopy signatures still demonstrated independent predictive signal. However, for the other pathways, phenocopy signatures generally outperformed DNA mutations (pathogenic or otherwise) in predicting response to targeted drugs across multiple agents and gene targets. These results are particularly impressive given that the phenocopy signatures were not directly trained to predict drug response, and instead appear to do so simply by virtue of their biological imperative, which is to identify phenocopies of DNA alterations.

### Sensitivity, specificity, PPV, and NPV of phenocopy signatures

**Fig. 4:**
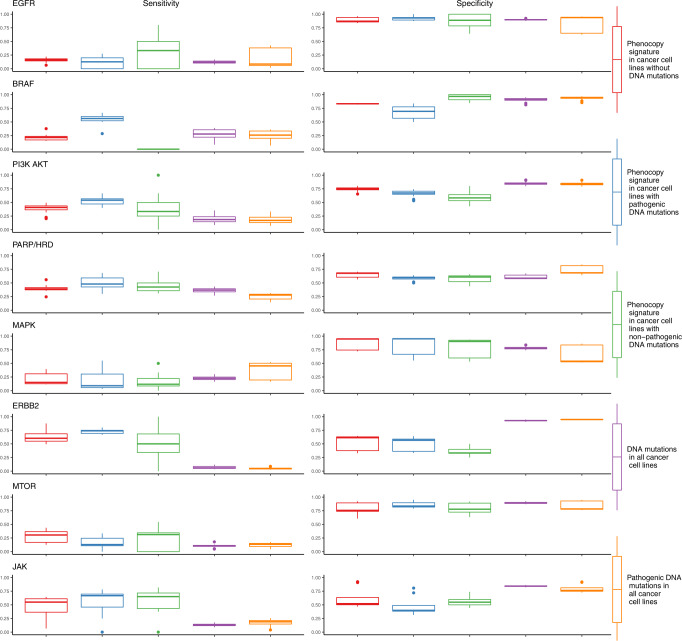
Sensitivity and specificity of phenocopy signatures. Sensitivity and specificity were calculated for each combination of drug and pathway. The top quartile in drug sensitivity for each drug was considered a responder. Each data point represents a single drug in a single dataset, with the three datasets represented by different shapes. Five separate conditions were investigated: (1) Phenocopy signature in cancer cell lines without DNA mutations, (2) Phenocopy signature in cancer cell lines with pathogenic DNA mutations, (3) Phenocopy signature in cancer cell lines with non-pathogenic DNA mutations, (4) DNA mutations in all cancer cell lines, and (5) Pathogenic DNA mutations in all cancer cell lines. Boxplot center line = median value, bounds of box (hinges) = interquartile range (IQR), bounds of whiskers = maximum of 1.5X IQR from the hinges.

**Fig. 5:**
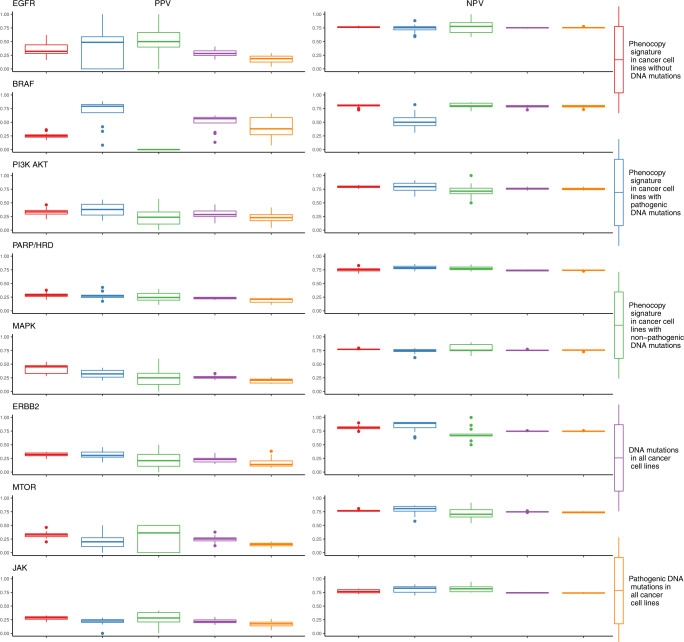
Positive predictive value (PPV) and negative predictive value (NPV) of phenocopy signatures. PPV and NPV were calculated for each drug and pathway. The top quartile in drug sensitivity for each drug was considered a responder. Each data point represents a single drug in a single dataset, with the three datasets represented by different shapes. Five separate conditions were investigated: (1) phenocopy signature in cancer cell lines without DNA mutations, (2) phenocopy signature in cancer cell lines with pathogenic DNA mutations, (3) phenocopy signature in cancer cell lines with non-pathogenic DNA mutations, (4) DNA mutations in all cancer cell lines, and (5) pathogenic DNA mutations in all cancer cell lines. Boxplot center line = median value, bounds of box (hinges) = interquartile range (IQR), bounds of whiskers = maximum of 1.5X IQR from the hinges.

Sensitivity, specificity, positive predictive value (PPV), and negative predictive value (NPV) are commonly used to evaluate clinical biomarkers. In our cell line models, we defined responders as the top quartile. Across all the drugs and pathways tested, 28% of cell lines with a mutation were classified as responders. When limited to pathogenic mutations, this percentage was similar at 26%. In cell lines without a mutation but were predicted to be a phenocopy, a slightly higher 31% were classified as responders, though the sensitivity in individual pathways was frequently higher. The sensitivity of the phenocopy signatures in mutation-negative cancer cell lines was on par with DNA alterations for EGFR, BRAF, and MAPK, and better than DNA alterations for PI3K-AKT, PARP/HRD, ERBB2, MTOR, and JAK. Oncogenic activation of *ERBB2* (gene encoding HER2) in particular is thought to be heavily influenced by amplification, and our results suggest that a mutational phenocopy signature may provide complementary information. The specificity of the phenocopy signatures was high across pathways in identifying responders in cell lines without DNA mutations (Fig. 4). The PPVs in cell lines without mutations are almost all improved compared to the results observed with DNA mutations, with the exception of BRAF in which the DNA mutations perform particularly well (Fig. 5). As with specificity, the NPVs are high for the phenocopy signatures across groups. These results confirm that the phenocopy signatures are successfully finding additional responders without DNA mutations with high specificity. While the sensitivity is not as high as the specificity, it is still comparable or better than DNA mutations alone.

### Clinical validation

**Fig. 6:**
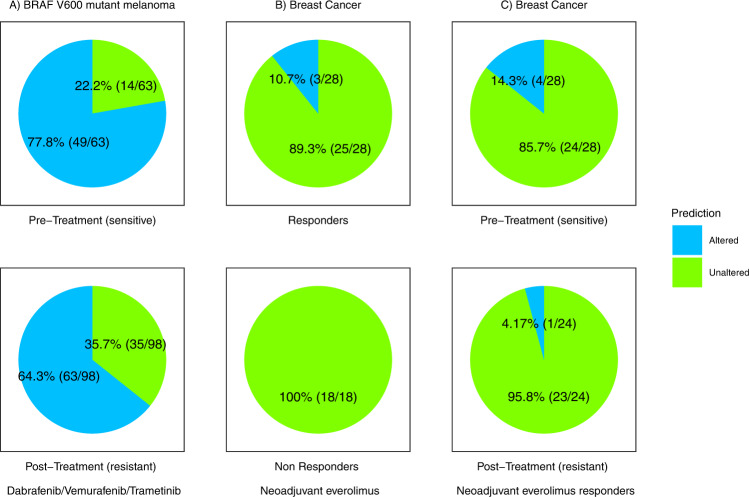
Clinical validation of phenocopy signatures. Our BRAF and mTOR phenocopy signatures were applied to BRAF-mutant melanoma (**A**) and breast cancer cohorts (**B**, **C**), respectively. Altered or unaltered status indicates the alteration status assigned by the BRAF/mTOR phenocopy signatures. Pre-treatment samples were considered sensitive, and post-treatment samples were considered resistant per the original datasets.

In addition to assessing our model in cell line datasets, we next sought to assess the efficacy of predicting drug response from a phenocopy signature in clinical data. We were able to identify several publicly available clinical cohorts that had treatment response and/or pre/post treatment resistance information for treatments specifically targeting the pathways of our phenocopy signatures. We first examined the BRAF pathway and identified three *BRAF*-mutant melanoma cohorts with gene expression data (GEO IDs: GSE50509, GSE65185, GSE99898) that were all treated with anti-BRAF therapies (dabrafenib, vemurafenib, trametinib)^42–44^. In all three cohorts, pre-treatment (sensitive) and post-treatment (resistant) samples were obtained from the same patients. Because the three cohorts were quite small, and similar in nature, we combined the results of all three. No additional DNA sequencing data were available for these cohorts. Our normalization approach and phenocopy signatures were applied without modification to each of the three cohorts. Overall, the majority (77.8%) of the pre-treatment (treatment-sensitive) samples were predicted to be BRAF mutation phenocopies, consistent with the fact that all the tumors were known to have *BRAF* mutations. Not all *BRAF* mutations are necessarily driver mutations. Thus, we would not expect the phenocopy predictions to match up exactly with the DNA mutations. The pre-treatment baseline phenocopy percentage is similar to the response rate of 68% to dabrafenib plus trametinib in the landmark COMBI-d and COMBI-v clinical trials^45^. However, this rate decreased to 64.3% in the post-treatment (treatment-resistant), with a borderline p-value of 0.0806 (Fig. 6A). This is consistent with our in-vitro data that the BRAF phenocopy signature predicts response to BRAF inhibitors, as the resistant tumors had a lower rate of phenocopies.

Everolimus is only approved in hormone-receptor positive HER2-negative advanced breast cancers. We next identified a cohort of ER + breast cancer patients (GSE119262) who were treated with neoadjuvant everolimus (which targets the mTOR pathway) followed by surgery^46^. In this cohort, both treatment response information and pre-treatment (sensitive) vs. post-treatment (resistant) samples were available. We first examined just the pre-treatment samples. While only a small number were predicted as mTOR mutation phenocopies, 100% of these responded (as defined in the original publication^46^) to anti-mTOR therapy compared to 75.8% of the non-mTOR phenocopy tumors (Fig. 6B). The overall phenocopy percentage was 8.7% in the pre-treatment samples, which is similar to the 9.5% response rate to everolimus plus exemestane shown in advanced hormone-receptor positive breast cancer in the landmark BOLERO-2 clinical trial^47^. When we further examined our phenocopy signature in pre-treatment and post-treatment samples, again none of the non-responder tumors (pre- or post-treatment) were predicted as phenocopies. In the responder tumors, there was a decrease in the rate of phenocopy tumors from 14.3% pre-treatment (sensitive) to 4.17% post-treatment (resistant; Fig. 6C). This is consistent with our in-vitro data which demonstrates that a phenocopy signature predicts response to mTOR inhibitors, as the post-treatment resistant tumors had a lower rate of phenocopies. While the small numbers are under-powered to detect statistical significance, the results are nonetheless consistent with our hypothesis.

## Discussion

Targeted therapies have shown great promise in treating a variety of cancer types, but to date only benefit a minority of cancer patients. A major reason is that targeted therapies perform optimally in patients whose specific tumors are uniquely dependent on the targeted pathway, which is currently assessed by identifying key driver mutations. The majority of patients lack a DNA alteration, and we do not currently have other biomarkers to identify additional patients who could benefit from these targeted treatments. With the creation of large pharmacogenomic databases^2,23,48^, most published efforts have been focused on specifically training molecular signatures to predict drug response^21–32^. Our phenocopy approach differs from this direct approach. Instead, we trained phenocopy signatures to identify the gene expression patterns that accompany common driver gene alterations in cancer. We then demonstrate that this indirect approach improves the ability to predict pan-cancer treatment response across eight oncogenic pathways compared to DNA mutation status alone. To our knowledge, this is the first report of the successful global application of a phenocopy strategy in predicting drug response in vitro and in clinical cohorts.

We show that in mutation-negative tumors, the phenocopy signatures can identify a subset that respond to targeted therapies with high specificity. These results suggest that phenocopy signatures add to clinically actionable mutations in predicting therapy response and could be used in clinical settings to identify mutation-negative patients who may benefit from targeted therapy with high specificity. While the sensitivity is not as high, it is comparable to DNA mutations alone and doubling the number of patients eligible for targeted therapies would represent an enormous clinical advancement. In addition, phenocopy signatures could also be used to help guide treatment decisions for patients with variants of unknown significance. Finally, most drug-biomarker indications are currently limited to specific cancer sites. Our training and validation cohorts are pan-cancer datasets, potentially allowing for a tremendous expansion of current targeted therapy indications across multiple cancer types.

The transcriptome-wide measure of gene expression via RNA-seq has been shown to be a useful metric to complement DNA alteration data in predicting drug responses in many contexts, given that gene expression provides a more functional snapshot of the cell’s phenotypic state compared to its genomic sequence. For example, gene expression-based RB1 loss signatures have been shown to be prognostic across cancer types (but have not been assessed regarding drug response)^49^, and Ras-pathway activation signatures have been shown to predict MEK inhibitor sensitivity^50^. Additionally, in a study examining the contributions of various molecular data types for assessing drug response, gene expression was found to be the most predictive metric across all cancer types when compared to gene mutation, copy number, methylation status, and tissue origin^22^. However, the same study found that combinations of data types were better at predicting drug response within cancer sub-types, highlighting the usefulness of combining DNA mutation status with RNA signatures for drug response predictions.

The primary limitation of this study is that the majority of drug response validation was based on cell lines, albeit in three large independent cohorts. We do have some clinical validation in public datasets, which were the only available cohorts with gene expression data from sensitive/resistant samples that we could identify. Nonetheless, further validation in larger clinical cohorts and trials is required. Clinical trials or cohorts of targeted therapies with transcriptome-wide RNA profiling are rare, though more recently have begun to have more use such as in the WINTHER trial (NCT01856296)^51^. This is partly because most commercial DNA sequencing panels do not include whole-transcriptome RNA-seq. Our study provides strong rationale for expanding clinical Next-Gen Sequencing to include RNA-seq, and provides a pan-cancer, platform-independent, phenocopy biomarker with which to select patients for inclusion in a next-generation clinical trial of targeted therapies in patients without driver DNA mutations. Indeed, these phenocopy signatures will be integrated into the planned biomarker-driven Alliance A032102 PREDICT trial in prostate cancer.

## Methods

We trained our phenocopy signatures on all protein-coding DNA alterations in the clinical TCGA dataset, which has minimal treatment response information. This is possible because we are not directly training on drug response, and our indirect approach has an added benefit in allowing us to save all cell line and clinical datasets with drug response for validation without having to worry about information leakage. Previous approaches directly training on cell line drug response face challenges in identifying suitable validation cohorts, as many of the cell lines overlap between different cell line datasets and clinical validation cohorts are rare.

### DNA mutation annotation

DNA mutations were annotated with Annovar^52^. Only protein sequence-altering mutations were included. Silent, splicing, intronic, upstream, and downstream mutations were excluded from our analysis. The rate of pathogenic (defined by ClinVar) non-coding mutations was rare (Supplementary Table 1). To identify mutations with stronger evidence for being pathogenic, ClinVar and various computational tools (SIFT, Polyphen-2 HVAR, Polyphen-2 HDIV, and FATHMM) were used. A sample was considered to have a pathogenic mutation if predicted by any of the computational tools or marked as pathogenic or likely pathogenic by ClinVar. A total of eight oncogenic signaling pathways with targeted drugs and mutations in the key driver genes were assessed (EGFR, BRAF, PI3K-AKT, PARP/HRD, ERBB2, mTOR, JAK, and MAPK). *EGFR* mutations were assessed for the EGFR pathway. *BRAF* mutations were assessed for the BRAF pathway. *PIK3CA*, *AKT1*, and *AKT2* mutations were assessed for the PIK3-AKT pathway. *BRCA1/2*, *ATM*, and *PARP1/2* mutations were assessed for the PARP/HRD pathway. *ERBB2* mutations were assessed for the ERBB2 pathway. *MTOR* mutations were assessed for the MTOR pathway. *JAK1/2/3* mutations were assessed for the JAK pathway. *MAPK11, MAPK12, MAPK13, MAPK14, MAPK3, MAPK1, MKNK1, MKNK2, MAP2K1, MAP2K2, MAPK8, MAPK9*, and *MAPK10* were assessed for the MAPK pathway. While amplifications and deletions are also important, we chose not to include these for training due to the lack of consistent thresholds for determining when a copy number change influences function, as well as the significant effects of tumor purity on copy number in the clinical samples.

### Phenocopy signature training

Prior to training the phenocopy signature we filtered each dataset to only include genes within the pathway of interest as determined by the Reactome^53^ database of gene pathways (Supplementary Table 2). For each gene pathway, we removed cancer types with an alteration rate below 5% from our TCGA training dataset. We then used a gradient tree boosting approach to train phenocopy signatures which predicted mutation status (true or false) based on RNA expression. Gradient tree boosting (e.g. XGboost) is an ensemble learning method where decision trees are constructed to minimize a differentiable loss function. This is done through a gradient descent algorithm where trees are iteratively fit to the direction of steepest descent of the loss function. XGboost builds upon ensemble tree models such as random forests by adding a regularization term, designed to control model complexity and avoid overfitting. We trained our signature on the TCGA dataset using the R XGboost package (version 1.4.0.1). XGboost offers a GPU-based implementation of gradient tree boosting that leverages a histogram algorithm to find candidate splits, which provides immense speed improvements. We applied this approach with a hinge loss function and used 10-fold cross validation to tune the depth and number of trees, with model accuracy assessed using Receiver Operator Curve (ROC) Area Under the Curve (AUC). A total of eight phenocopy signatures were trained, one for each oncogenic signaling pathway, and were locked prior to independent validation.

### Independent validation of the phenocopy signatures in GDSC, CCLE, and DepMap

Each of the eight oncogenic pathways were tested in the GDSC, CCLE, and DepMap cohorts. Response for drugs specifically targeting each pathway was assessed per pathway as above. Mutations were assessed as above. The phenocopy signatures were applied without modification to the GDSC/CCLE/DepMap datasets and resulted in predicted mutation status to identify phenocopies. GDSC, CCLE, and DepMap sets were validated independently. As all three study cancer cell lines and anti-cancer drugs, there is overlap. However, as the experiments were done at different times with different techniques, we chose to investigate them as independent datasets.

### Statistical approach

To compare whether the phenocopy signatures improved the ability to predict response to targeted therapies, we created linear models with the actual drug response (Z-score for the IC-50 for GDSC, the ActArea for CCLE, AUC for DepMap) as the dependent variable, and the actual and predicted mutation status as the independent variables. For the CCLE, IC-50 was not utilized due to 55% of all IC-50 values being the maximum tested concentration of 8 μM, therefore activity area (ActArea) was used, where a higher ActArea corresponds to increased sensitivity^23^. For DepMap, 69% of IC-50 values were reported as NA, thus AUC was used as a measure of drug response. While using the same drug response metrics across datasets would have been ideal, diverse measures can provide complementary information even with the same cell lines/drugs and ensure our results are independent of the dataset. Model fit was determined using the ordinary least squares approach. Coefficients from the model indicate how strongly the actual and predicted alteration statuses contribute to drug response. We also performed a likelihood-ratio test using the chi-square statistic (*χ*^2^) to compare a single parameter model (mutation status alone) and a two parameter (mutation status and the phenocopy signature) model in order to assess if the phenocopy signature was significantly adding to DNA mutations alone in predicting drug response. Because the models are nested, the degrees of freedom equal the difference in the number of free parameters in the two models. Thus, the two parameter model is a significant improvement over the single parameter model if the observed *χ*^2^ statistic is >4.5 corresponding to a Benjamini-Hochberg FDR multiple testing corrected p-value cutoff of 0.05.

### Sensitivity and specificity of the phenocopy signature in predicting drug response

We next assessed the sensitivity and specificity of the phenocopy signatures. Because drug response was a continuous variable in our cell line datasets, we stratified “responders” and “non-responders” based on the top quartile vs. the bottom three quartiles^54^. To better understand the performance in the context of DNA mutations, we considered three subgroups: (1) cell lines without mutations, (2) cell lines with mutations that were not predicted to be pathogenic (e.g. unknown clinical significance) and (3) cell lines with mutations predicted to be pathogenic. We then compared this to the sensitivity and specificity of mutations alone, or pathogenic mutations alone.

### Reporting summary

Further information on research design is available in the Nature Research Reporting Summary linked to this article.

## Supplementary information

Supplemental Data

Reporting Summary Checklist

## Data Availability

Processed DNA and RNA sequencing data from the Cancer Genome Atlas (TCGA) were downloaded using the UCSC Xena browser (xena.ucsc.edu). Processed DNA and RNA sequencing data and drug response data for the Genomic of Drug Sensitivity in Cancer (GDSC)^48^ were downloaded from the GDSC website (www.cancerrxgene.org). Processed DNA and RNA sequencing data and drug response data for the Cancer Cell Line Encyclopedia (CCLE)^23^ were downloaded from the CCLE website (portals.broadinstitute.org/ccle). The Cancer Dependency Map (DepMap)^2^ shares the same cell lines and therefore DNA and RNA sequencing data as the CCLE, but independently tests treatment response, and these were obtained from the DepMap website (depmap.org). As recommended by DepMap, the MTS010 dataset was used for drug response data. Datasets were then filtered to only include the genes present in all three datasets. To allow comparability between groups, gene expression was normalized as previously described^55^. Gene expression was treated as a continuous variable throughout the study. DNA mutation calls for TCGA, CCLE (including DepMap), and GDSC, were used as described in each dataset. Clinical datasets^42–44,46^ were downloaded from the Gene Expression Omnibus (www.ncbi.nlm.nih.gov/geo) with the following accession numbers: GSE50509, GSE65185, GSE99898, and GSE119262.

## Code availability

Details on the R code used in this manuscript will be available for reasonable non-commercial academic purposes upon request.
