# Supplementary material for: Identification of phenocopies improves prediction of targeted therapy response over DNA mutations alone
Source: NPJ Genom Med. 2022 Oct 17;7:58. doi: 10.1038/s41525-022-00328-7 (PMC9576758; doi:10.1038/s41525-022-00328-7)
Supplement: Supplementary file 1 — Supplemental Data [file 41525_2022_328_MOESM1_ESM.pdf]

SUPPLEMENTAL FIGURES

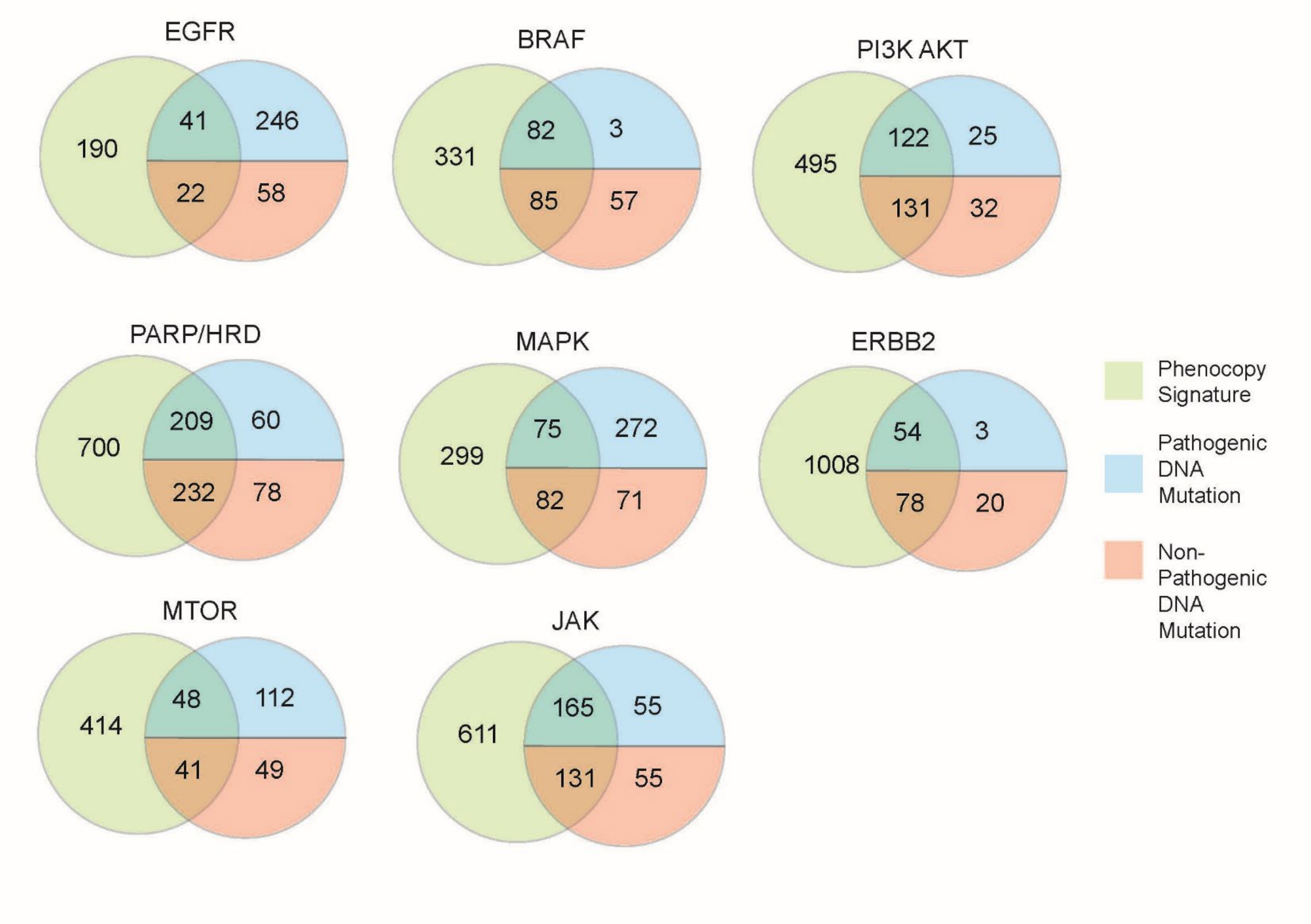

Supplementary Figure 1. Phenocopy signature predictions versus DNA mutations.
Venn diagrams depict the number of cell lines assigned as phenocopies compared to actual DNA mutations across the eight pathways tested. DNA mutations are further divided into those that are annotated as pathogenic by ClinVar or various computational tools and those that have unknown significance.

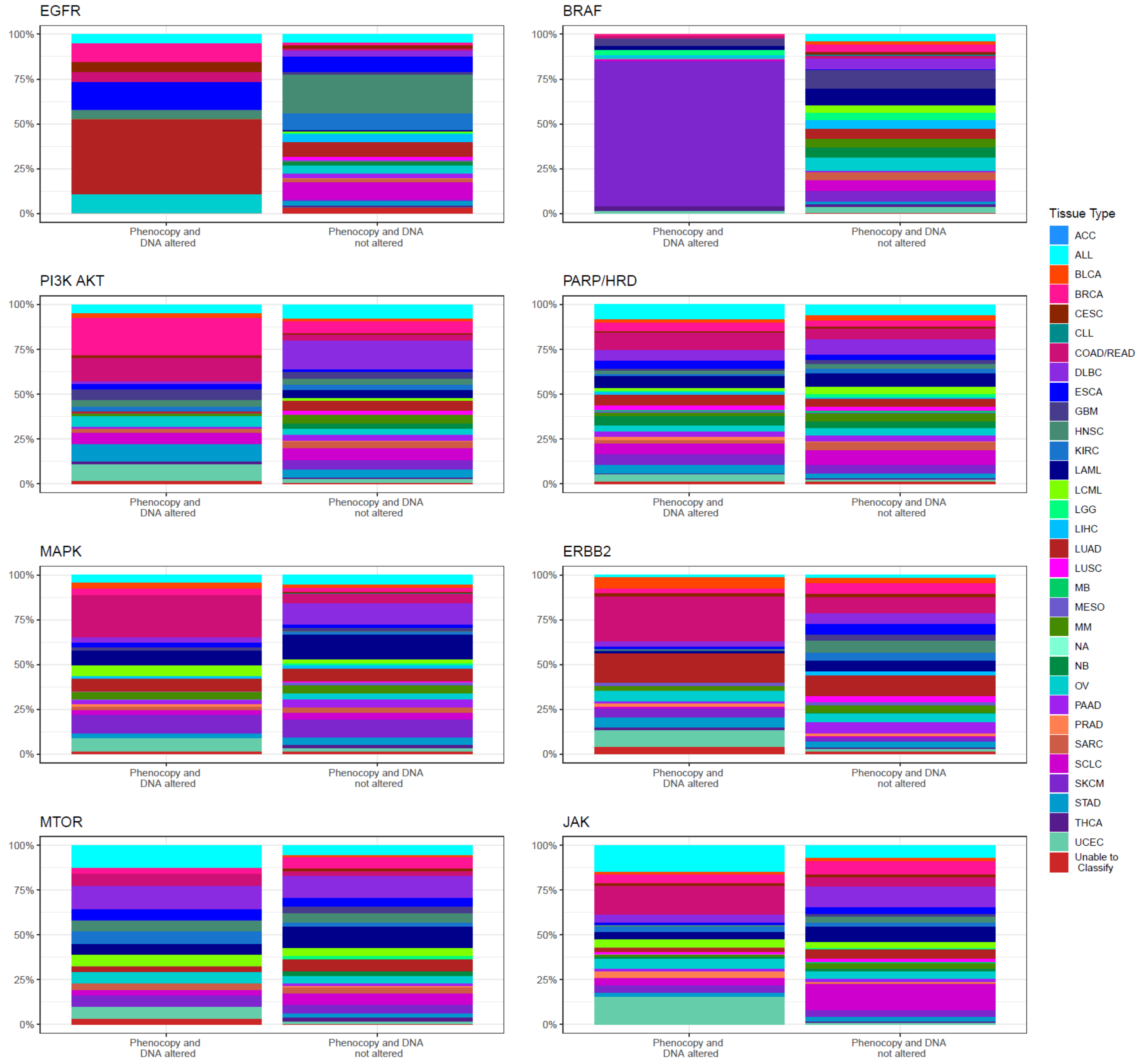

Supplementary Figure 2: Tumor type breakdown across phenocopies in our cell line validation cohorts.

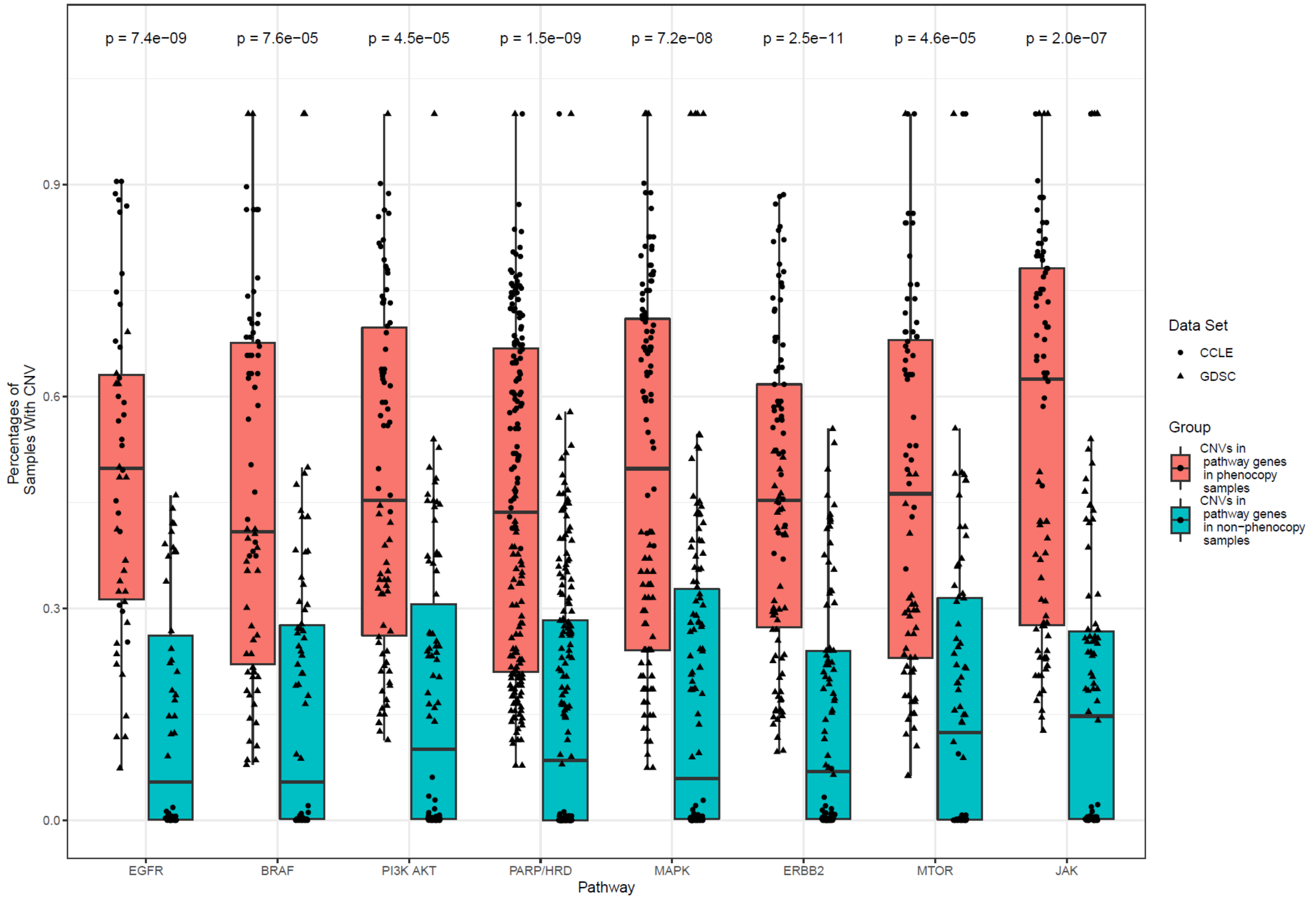

Supplementary Figure 3: Boxplot showing the rates of CNV in genes in each pathway, excluding the mutated genes on which the phenocopy signatures were trained (e.g. excluding BRAF in the BRAF pathway). Each point represents a gene. We used both our cell line validation datasets (GDSC, CCLE), and defined amplification/deletion with GISTIC thresholds of ≥2 or ≤-2 in the GDSC, and the equivalent CN thresholds in CCLE. Only samples without a mutation in the driver genes are included (e.g. only BRAF mutation negative samples for the BRAF pathway). Boxplots show median values and interquartile ranges.

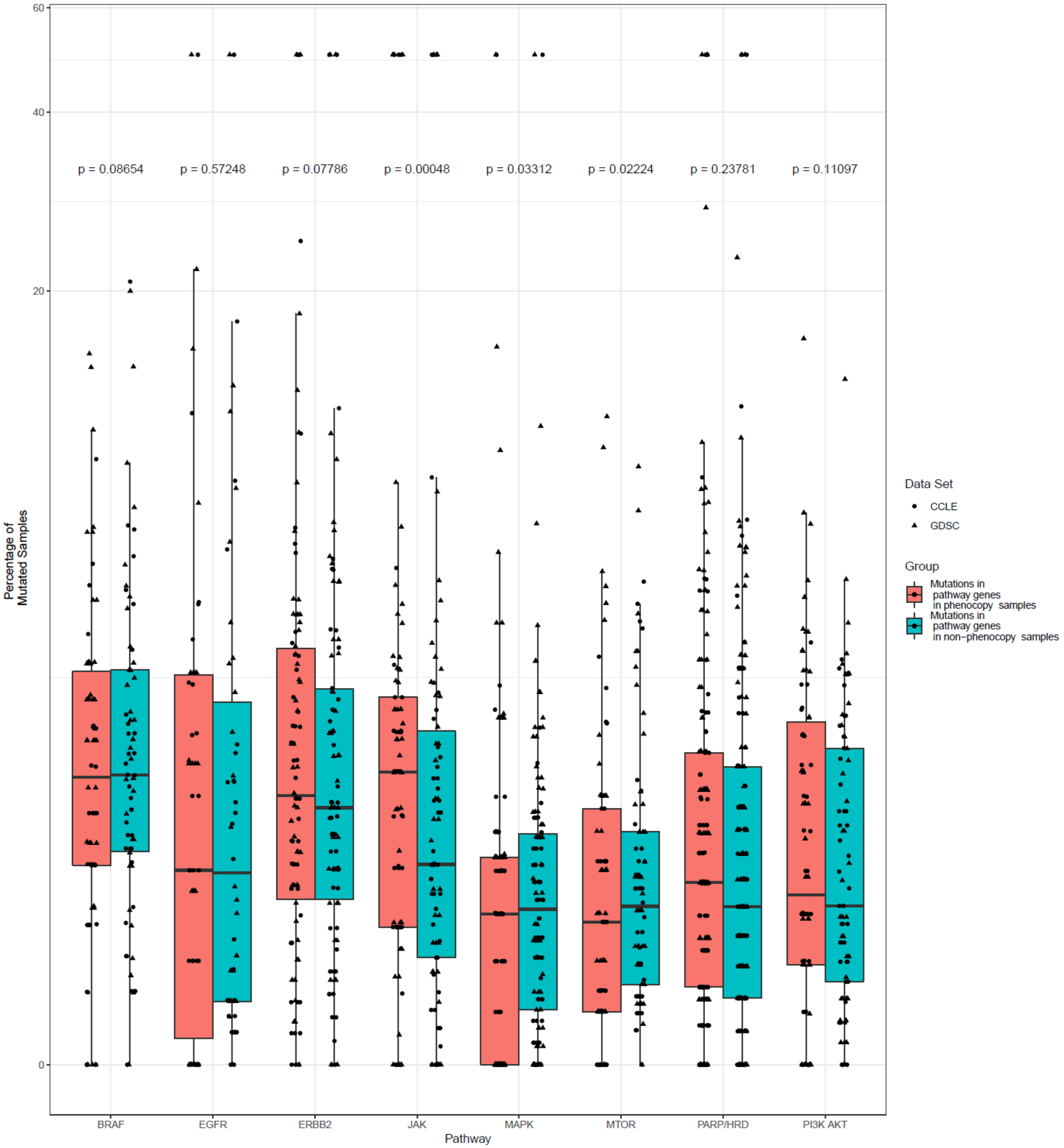

Supplementary Figure 4: Boxplot showing the rates of mutation in genes in each pathway, excluding the mutated genes on which the phenocopy signatures were trained (e.g. excluding BRAF in the BRAF pathway). Each point represents a gene. We used both our cell line validation datasets (GDSC, CCLE). Only samples without a mutation in the driver genes are included (e.g. only BRAF mutation negative samples for the BRAF pathway). Boxplots show median values and interquartile ranges.

SUPPLEMENTAL TABLES

Supplementary Table 1: ClinVar pathogenic non-coding mutations across pathways

Pathway	Samples
EGFR	0
BRAF	0
PI3K AKT	0
PARP/HRD	7
MAPK	0
ERBB2	0
MTOR	0
JAK	0

Supplementary Table 2: pathway genes

EGFR	BRAF	PI3K_AKT	PARP/HRD	MAPK	ERBB2	MTOR
HSP90AA1	BRAF	GAB2	RAD52	MAP2K3	MATK	RRAGD
CDC37	RAP1B	PIK3CB	BRCA1	MAPK9	FYN	EIF4B
AREG	KSR1	FGFR2	ERCC1	CUL1	ERBB3	STRADB
GAB1	ARRB1	FGFR3	RFC1	TAB2	RHOA	RRAGB
CBL	HRAS	FGF10	RFC2	MAP2K4	PTPN18	PPM1A
HBEGF	MARK3	FGF22	RAD51	MEF2A	HSP90AA1	CAB39L
SOS1	ARRB2	FGF4	POLD1	RPS6KA2	PTK6	TSC2
PIK3CA	CSK	FGFR1	RNF4	FBXW11	STUB1	EEF2K
PLCG1	MAP2K1	PIK3C3	TP53BP1	MAP2K7	AKT2	AKT2
EREG	RAF1	FGF20	TIPIN	MEF2C	CDC37	RHEB
KRAS	MAPK1	FLT3LG	SIRT6	MAPK1	GAB1	PRKAG2
EGF	NRAS	TRIB3	POLD3	TAB1	HBEGF	RPS6KB1
RPS27A	VWF	FGF9	PALB2	RPS6KA5	SOS1	LAMTOR3
PIK3R1	ARAF	AKT2	UIMC1	MAPK3	AKT3	PRKAB1
EGFR	MAPK3	FGF8	CLSPN	RIPK2	PIK3CA	EIF4G1
UBC	RAP1A	GAB1	ABL1	IKBKB	PLCG1	PRKAG3
SHC1	VCL	FGF6	POLE2	PPP2CB	EREG	LAMTOR2
TGFA	IQGAP1	FGF1	RBBP8	VRK3	PTPN12	RRAGC
UBB	YWHAB	FGF23	UBE2I	PPP2R1A	DIAPH1	STK11
HRAS	TLN1	PIK3CA	NBN	NOD1	KRAS	PRKAB2
BTC	FN1	FLT3	PIAS4	MAPK8	USP8	PRKAA1
GRB2	PEBP1	KL	BABAM1	MAP3K8	EGF	LAMTOR5
EPGN	ACTG1	KLB	RPA3	DUSP3	ERBB2	CAB39
NRAS	ACTB	FGF5	POLD2	MAP2K6	GRB7	RPS6
UBA52	CNKSR1	FGF2	RAD51C	NFKB1	AKT1	RPTOR
	APBB1IP	FGF7	RAD51AP1	MAPK10	RPS27A	AKT1
	SRC	PDPK1	RFC5	MAPK14	PIK3R1	LAMTOR1
	ITGB3	PIK3R1	TIMELESS	PPP2R5D	EGFR	EIF4E
	ITGA2B	PDE3B	RNF8	SKP1	UBC	RRAGA
	CNKSR2	FGF18	RAD1	PPP2CA	PRKCA	PRKAA2
	KSR2	FGF17	RAD50	MAPKAPK3	NRG1	TSC1
	FGG	THEM4	POLE4	ATF2	NRG2	YWHAB
	FGA	FGFR4	SUMO1	RPS6KA1	SHC1	MLST8
	FGB	FGF19	RPA2	CREB1	MEMO1	SLC38A9
	KRAS	FRS2	POLK	DUSP4	PRKCD	PRKAG1
		IRS1	CDK2	ATF1	CUL5	EIF4EBP1
		GRB2	XRCC3	ELK1	NRG4	LAMTOR4
		PTPN11	HERC2	IRAK2	UBB	MTOR
		IRS2	RPA1	MAP3K7	PRKCE	AKT1S1
		PIK3R4	PCNA	PPP2R1B	HRAS	STRADA
		FGF16	CCNA1	DUSP6	BTC	
			RFC3	RPS27A	YES1	
			HUS1	UBC	GRB2	
			BRIP1	TAB3	ERBB4	
			MDC1	MAPKAPK2	RNF41	
			DNA2	DUSP7	NRG3	

BARD1	BTRC	SRC
BRCA2	MAPK7	NRAS
RPS27A	NOD2	UBA52
CCNA2	TNIP2	
POLE3	MAP2K1	
ATM	UBB	
CHEK1	FOS	
PPP4C	TRAF6	
UBC	RPS6KA3	
RAD9B	JUN	
RAD17	UBE2N	
EME1	IRAK1	
PPP4R2	MAPK11	
TOPBP1	CHUK	
RFC4	UBA52	
RNF168	UBE2V1	
ATRIP	IKBKG	
SPIDR		
WRN		
UBE2V2		
UBB		
POLH		
RHNO1		
RAD9A		
MUS81		
KAT5		
EXO1		
ATR		
POLD4		
ERCC4		
RMI2		
POLE		
TOP3A		
UBE2N		
GEN1		
RMI1		
RAD51B		
RAD51D		
BRCC3		
SUMO2		
SLX4		
XRCC2		
BLM		
EME2		
UBA52		
RTEL1		
