# Supplementary material for: Identification of phenocopies improves prediction of targeted therapy response over DNA mutations alone
Source: NPJ Genom Med. 2022 Oct 17;7:58. doi: 10.1038/s41525-022-00328-7 (PMC9576758; doi:10.1038/s41525-022-00328-7)
Supplement: Supplementary file 2 — Reporting Summary Checklist [file 41525_2022_328_MOESM2_ESM.pdf]

nature portfolio

Corresponding author(s): Shuang Zhao

Last updated by author(s): Jul 13, 2022

Reporting Summary

Nature Portfolio wishes to improve the reproducibility of the work that we publish. This form provides structure for consistency and transparency in reporting. For further information on Nature Portfolio policies, see our Editorial Policies and the Editorial Policy Checklist.

Statistics

For all statistical analyses, confirm that the following items are present in the figure legend, table legend, main text, or Methods section.

n/a	Confirmed	
☐	☒	The exact sample size (*n*) for each experimental group/condition, given as a discrete number and unit of measurement
☒	☐	A statement on whether measurements were taken from distinct samples or whether the same sample was measured repeatedly
☐	☒	The statistical test(s) used AND whether they are one- or two-sided<br>*Only common tests should be described solely by name; describe more complex techniques in the Methods section.*
☐	☒	A description of all covariates tested
☐	☒	A description of any assumptions or corrections, such as tests of normality and adjustment for multiple comparisons
☒	☐	A full description of the statistical parameters including central tendency (e.g. means) or other basic estimates (e.g. regression coefficient) AND variation (e.g. standard deviation) or associated estimates of uncertainty (e.g. confidence intervals)
☐	☒	For null hypothesis testing, the test statistic (e.g. *F*, *t*, *r*) with confidence intervals, effect sizes, degrees of freedom and *P* value noted<br>*Give P values as exact values whenever suitable.*
☒	☐	For Bayesian analysis, information on the choice of priors and Markov chain Monte Carlo settings
☒	☐	For hierarchical and complex designs, identification of the appropriate level for tests and full reporting of outcomes
☐	☒	Estimates of effect sizes (e.g. Cohen's *d*, Pearson's *r*), indicating how they were calculated

Our web collection on statistics for biologists contains articles on many of the points above.

Software and code

Policy information about availability of computer code

Data collection	No software was used
Data analysis	R 4.0.4

For manuscripts utilizing custom algorithms or software that are central to the research but not yet described in published literature, software must be made available to editors and reviewers. We strongly encourage code deposition in a community repository (e.g. GitHub). See the Nature Portfolio guidelines for submitting code & software for further information.

Data

Policy information about availability of data

All manuscripts must include a data availability statement. This statement should provide the following information, where applicable:

- Accession codes, unique identifiers, or web links for publicly available datasets
- A description of any restrictions on data availability
- For clinical datasets or third party data, please ensure that the statement adheres to our policy

Processed DNA and RNA sequencing data from the Cancer Genome Atlas (TCGA) were downloaded using the UCSC Xena browser (xena.ucsc.edu). Processed DNA and RNA sequencing data and drug response data for the Genomic of Drug Sensitivity in Cancer (GDSC)46 were downloaded from the GDSC website (www.cancerrxgene.org). Processed DNA and RNA sequencing data and drug response data for the Cancer Cell Line Encyclopedia (CCLE)23 were downloaded from the CCLE website (portals.broadinstitute.org/ccle). The Cancer Dependency Map (DepMap)2 shares the same cell lines and therefore DNA and RNA sequencing data

as the CCLE, but independently tests treatment response, and these were obtained from the DepMap website (depmap.org). As recommended by DepMap, the MTS010 dataset was used for drug response data. Datasets were then filtered to only include the genes present in all three datasets. To allow comparability between groups, gene expression was normalized as previously described52. Gene expression was treated as a continuous variable throughout the study. DNA mutation calls for TCGA, CCLE (including DepMap), and GDSC, were used as described in each dataset. Clinical datasets42–45 were downloaded from the Gene Expression Omnibus (www.ncbi.nlm.nih.gov/geo) with the following accession numbers: GSE50509, GSE65185, GSE99898, and GSE119262.

Human research participants

Policy information about studies involving human research participants and Sex and Gender in Research.

Reporting on sex and gender	Not applicable, only publicly available de-identified data were used.
Population characteristics	Not applicable, only publicly available de-identified data were used.
Recruitment	Not applicable, only publicly available de-identified data were used.
Ethics oversight	Not applicable, only publicly available de-identified data were used.

Note that full information on the approval of the study protocol must also be provided in the manuscript.

Field-specific reporting

Please select the one below that is the best fit for your research. If you are not sure, read the appropriate sections before making your selection.

☒ Life sciences ☐ Behavioural & social sciences ☐ Ecological, evolutionary & environmental sciences

For a reference copy of the document with all sections, see nature.com/documents/nr-reporting-summary-flat.pdf

Life sciences study design

All studies must disclose on these points even when the disclosure is negative.

Sample size	Not applicable, only publicly available de-identified data were used.
Data exclusions	No data were excluded.
Replication	Not applicable, only publicly available de-identified data were used.
Randomization	Not applicable, only publicly available de-identified data were used.
Blinding	Not applicable, only publicly available de-identified data were used.

Reporting for specific materials, systems and methods

We require information from authors about some types of materials, experimental systems and methods used in many studies. Here, indicate whether each material, system or method listed is relevant to your study. If you are not sure if a list item applies to your research, read the appropriate section before selecting a response.

Materials & experimental systems

n/a	Involved in the study	
☒	☐	Antibodies
☒	☐	Eukaryotic cell lines
☒	☐	Palaeontology and archaeology
☒	☐	Animals and other organisms
☒	☐	Clinical data
☒	☐	Dual use research of concern

Methods

n/a	Involved in the study	
☒	☐	ChIP-seq
☒	☐	Flow cytometry
☒	☐	MRI-based neuroimaging

March 2021
